# Refinement of 16S rRNA gene analysis for low biomass biospecimens

**DOI:** 10.1038/s41598-021-90226-2

**Published:** 2021-05-24

**Authors:** Remy Villette, Gaelle Autaa, Sophie Hind, Johanna B. Holm, Alicia Moreno-Sabater, Martin Larsen

**Affiliations:** 1 Inserm UMR-S1135, Centre d’Immunologie et des Maladies Infectieuses (CIMI-Paris), 91 bd. de l’Hôpital, 75013 Paris, France; 2 Sorbonne Universités, UPMC Univ Paris 06, CR7, Centre d’Immunologie et des Maladies Infectieuses (CIMI-Paris), Hôpital Pitié-Salpêtrière, 83 bd. de l’Hôpital, 75013 Paris, France; 3 Department of Microbiology and Immunology, Institute for Genome Sciences, University of Maryland School of Medicine, Baltimore, MD USA; 4 Département D’Immunologie, AP-HP, Groupement Hospitalier Saint Louis, 75010 Paris, France

**Keywords:** Next-generation sequencing, Microbiome

## Abstract

High-throughput phylogenetic 16S rRNA gene analysis has permitted to thoroughly delve into microbial community complexity and to understand host-microbiota interactions in health and disease. The analysis comprises sample collection and storage, genomic DNA extraction, 16S rRNA gene amplification, high-throughput amplicon sequencing and bioinformatic analysis. Low biomass microbiota samples (e.g. biopsies, tissue swabs and lavages) are receiving increasing attention, but optimal standardization for analysis of low biomass samples has yet to be developed. Here we tested the lower bacterial concentration required to perform 16S rRNA gene analysis using three different DNA extraction protocols, three different mechanical lysing series and two different PCR protocols. A mock microbiota community standard and low biomass samples (10^8^, 10^7^, 10^6^, 10^5^ and 10^4^ microbes) from two healthy donor stools were employed to assess optimal sample processing for 16S rRNA gene analysis using paired-end Illumina MiSeq technology. Three DNA extraction protocols tested in our study performed similar with regards to representing microbiota composition, but extraction yield was better for silica columns compared to bead absorption and chemical precipitation. Furthermore, increasing mechanical lysing time and repetition did ameliorate the representation of bacterial composition. The most influential factor enabling appropriate representation of microbiota composition remains sample biomass. Indeed, bacterial densities below 10^6^ cells resulted in loss of sample identity based on cluster analysis for all tested protocols. Finally, we excluded DNA extraction bias using a genomic DNA standard, which revealed that a semi-nested PCR protocol represented microbiota composition better than classical PCR. Based on our results, starting material concentration is an important limiting factor, highlighting the need to adapt protocols for dealing with low biomass samples. Our study suggests that the use of prolonged mechanical lysing, silica membrane DNA isolation and a semi-nested PCR protocol improve the analysis of low biomass samples. Using the improved protocol we report a lower limit of 10^6^ bacteria per sample for robust and reproducible microbiota analysis.

## Introduction

The human body is colonized by complex microbial communities at most external body sites. We are now able to scrutinize the composition of microbiota communities from various body sites with low cost high-throughput sequencing technology. Except for some immune cells, aneuploid cells and cells with mitotic defects human cells can be considered genetically identical, contrary to the microbiota, which is genetically very diverse. This results in large phenotypic variations among microbes. Most notable is variation in microbial cell membrane (e.g. gram positive and negative), cell morphology (e.g. flagella, pili etc.) and cell–cell interaction (e.g. planktonic, cocci packets, biofilms etc.). Stool sample collection, storage, DNA extraction, PCR amplification, sequencing technology and bioinformatic analysis have been shown to dramatically modify microbiota analytical outcomes. These variations are most likely a result of genotypic and phenotypic variation of microbiota. To ensure reliability and reproducibility of microbiome studies it is crucial to develop a standardizable protocol, which optimize sensitivity and specificity of phylogenetic microbiota analysis. Genotypic bias should be reduced by better primer design and bioinformatic analysis and phenotypic bias should be addressed by employing DNA extraction protocols less sensitive to variability in microbial cell resistance to lysis. In absence of a globally accepted standardized protocol, studies so far employ different experimental protocols and analytical pipelines. Sample storage practice^1^ and freeze–thaw cycles^2^ were found to profoundly affect microbiome analysis. Extraction protocols have been shown to affect DNA yields^3,4^ and relative abundance within samples, and even an under-representation of dominating phyla in infant gut samples (Actinobacteria phyla^5^), resulting in a major bias between studies^3,6,7^. Choice of 16S rRNA gene variable region and associated primer design equally affect phylogenetic coverage^5,8–10^.

Recently, microbiota from scarcely colonized body sites gained interest, such as skin, lung or even breastmilk microbiomes as well as neonate infant gut microbiota or cell sorted microbiota. Importantly, such samples contain fewer bacteria than gut microbiota from adult donors, which has thus far been the most studied type of biospecimen. Only few studies have evaluated if classic 16S rRNA gene analysis protocols are appropriate for low biomass biospecimens^11–14^. This raises the question of feasibility and accuracy of low biomass microbiota studies; for instance the existence of a commensal community in utero is still a topic of debate^15–20^. Low microbial biomass samples could be strongly impacted by bacterial contaminants inherent of work environment^21,22^ or experimental artifacts. Therefore, determining the lower limit of microbiota material required for effective representative analysis is becoming mandatory. We already showed that bacterial concentration was a limiting factor for whole-genome shot-gun analysis^23^. Indeed, samples with less than 10^7^ microbes result in biased microbiome analysis. Whole genome shot-gun sequencing is therefore less suitable for the analysis of very low biomass microbiota. 16S rRNA gene amplicon sequencing benefit from PCR amplification, which makes it particularly adapted for analysis of low biomass microbiota samples. Whereas PCR can overcome the limitations associated with sample biomass, successive PCR amplification cycles may severely bias the analysis.

Finally, bioinformatic pipelines also introduce variations. Indeed, taxonomy assignment (UPARSE^24^, MOTHUR^25^, DADA2^26^), reference databases (Greengenes, SILVA, RDP and NCBI), depth normalization^27,28^ and distance algorithms are non-consensual and therefore introduce additional discrepancies between studies. Sinha and colleagues showed that bioinformatics contributed the least in comparison to extraction protocols or sequencing protocols^6^. We therefore decided not to assess the effect of bioinformatics processing in the present study.

Here, we propose a 16S rRNA gene sequencing protocol optimized for low biomass biospecimens. We focused the optimization on identifying a DNA extraction protocol minimally affected by microbial cell resistance to lysis as well as a PCR amplification protocol with minimal bias of downstream phylogenetic assignment. Optimizations were effectuated on donor stools and a commercial mock microbial community standard. Three different DNA extraction protocols and two PCR protocols were evaluated. Additionally, we used the corresponding genomic DNA commercial standard, which excludes DNA extraction bias, to evaluate the isolated effect of different PCR protocols. We demonstrate that sample biomass is the primary limiting step for microbiome analysis. We demonstrate that optimized DNA extraction and PCR protocols resulted in a tenfold improvement in sensitivity. We conclude that microbiota composition analysis using our optimized experimental protocol is robust and repetitive for samples containing as little as 10^6^ microbes.

## Results

### 16S rRNA gene sequencing is limited by sample biomass

**Figure 1:**
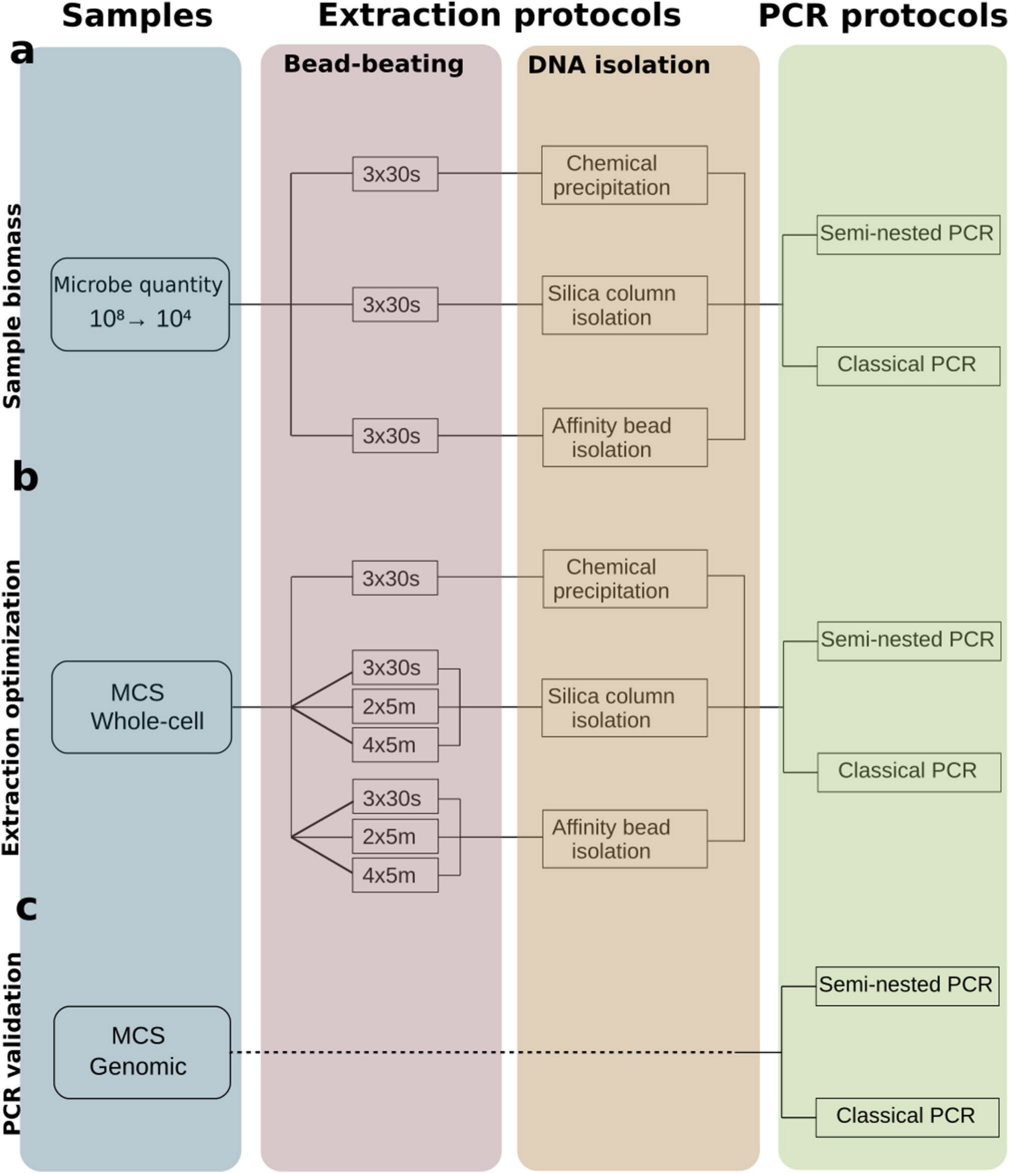
Summary of technical approaches presented in this paper. (**a**) Protocol for low biomass healthy donor stool samples containing 10^8^, 10^7^, 10^6^, 10^5^ and 10^4^ microbes. (**b**) Protocol tested on whole-cell mock microbial community standard (MCS). (**c**) PCR protocols used on genomic DNA mock microbial community standard. Figures were generated using R^39^.

Stools from two healthy donors were selected to assess sample biomass limit, below which 16S rRNA gene sequencing loses the ability to correctly represent microbiota composition. To this end we conducted serial dilutions to prepare samples of 10^8^, 10^7^, 10^6^, 10^5^ and 10^4^ microbes, which were extracted with three different extraction protocols. 16S rRNA genes were then amplified using two different PCR protocols, a semi-nested PCR (coined Nested) and a standard PCR (coined Standard). From the three different DNA extraction protocols, only the Zymobiomic’s Miniprep (MP) kit extracted genomic DNA from which the V3-V4 16S rRNA gene fragment could be amplified for all microbial dilutions. Chemical precipitation (CP) and Magbeads (MB) reached their limits for microbial quantities below 10^7^ and 10^5^ microbes, respectively (data not shown). Based on these results MP seems to perform better for low biomass samples than CP and MB. We therefore chose to sequence only low biomass samples extracted with MP (Fig. 1a).

Low microbial biomass samples, such as biopsies, swabs etc., contains considerable amounts of human DNA, which could cause off-target amplification of e.g. mitochondrial 16S rRNA genes. To exclude this potential bias, human DNA from whole blood of a healthy volunteer was extracted and amplified. No DNA amplification was observed for human DNA, including mitochondrial DNA (data not shown). This result is in line with *pairwise alignment*, which shows less than 1% homology between relevant PCR primers and human mitochondrial 16S rRNA genes.

In the absence of a theoretical reference microbial composition, we consider that the analysis of high microbial biomass samples (10^8^ microbes) provides the least biased microbial composition. Therefore, higher biomass samples will be used as reference point.

Low biomass samples produced fewer sequence reads leading to an important variability in sequencing depth (range: 1686–38,488 reads, Supplementary Fig. 1a). The design of our study requires that we analyze low biomass samples despite low sequence read counts. To evaluate potential bias caused by sequencing depth variation we conducted three different normalization protocols: (1) no rarefaction, (2) rarefaction to the sample with fewest sequence reads (1686 reads) and (3) rarefaction to 10,000 reads for the samples with at least 10,000 reads but non-rarefied retention of samples with less than 10,000 reads. The first analysis was conducted based on a hypothesis that low sequencing depth should be considered a consequence of suboptimal sample biomass, and thus a bias that should be considered when identifying lower biomass limit for 16S rRNA gene analysis. The second approach consisted in removing the sequencing depth bias to ensure that the effect witnessed in this paper was not a residual bias from sequencing depth differences. Finally, we decided to limit the sequencing depth to a maximum of 10,000 reads, which correspond to the theoretic number of cells from the least concentrated sample (10^4^). In doing so we ensure that low and high biomass samples are all theoretically able to represent the full extent of microbial diversity. All approaches resulted in the same findings and are available in the R code.

**Figure 2:**
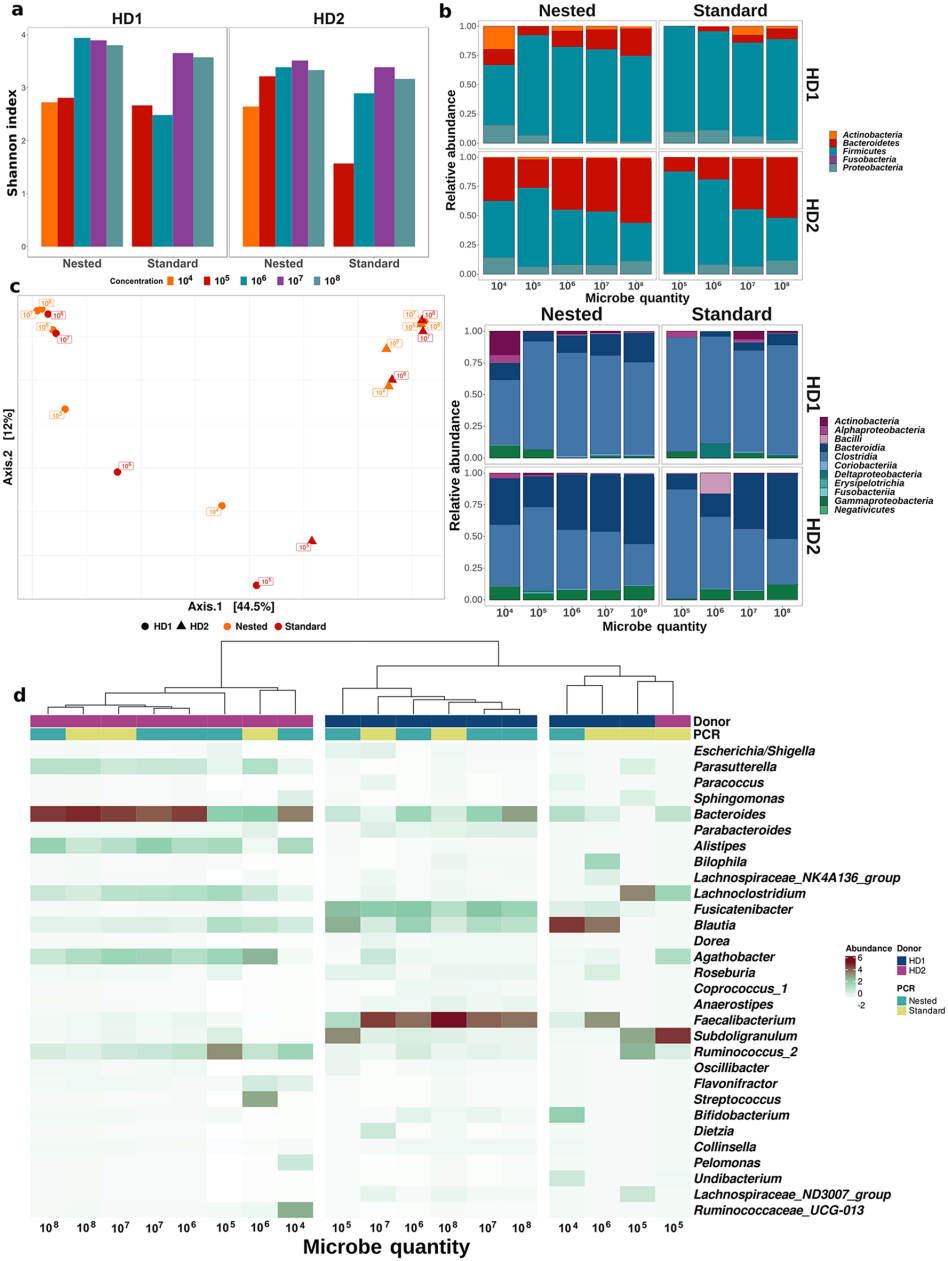
Effect of low biomass and PCR protocols on the representation of microbiota composition. (**a**) Alpha-diversity of low biomass samples. (**b**) Microbial composition at Phylum (upper panel) and Class (lower panel) phylogenetic level. (**c**) Principal coordinate analysis (PCoA) of samples using Bray–Curtis distance. Samples are labeled based on their cell content, shape represents donors and colors represent PCR protocols for each sample. (**d**) Heatmap of the top 30 most abundant genera, clustered using Bray–Curtis distance. Donor origin is represented by the first color layer and PCR protocols are represented by the second color layer. Of note, all samples were exposed to the same DNA extraction protocol (bead-beating for 3 × 30 s and purified on silica filters). Figures were generated using R^39^.

Both alpha diversity and species (amplicon sequence variant, ASV) richness increased with sample biomass, reaching maximal diversity at 10^6^ microbes. Interestingly, Nested PCR displayed a tendency for an overall higher alpha diversity compared to Standard PCR (*p* = 0.075, paired Student test). Maximum alpha diversity was reached for biomasses of 10^5^–10^6^ and 10^6^–10^7^ microbes for Nested and Standard PCR, respectively (Fig. 2a). Microbial composition at lower biomass is better preserved for healthy donor 2 compared to healthy donor 1. In all cases Nested PCR was able to correctly describe samples with tenfold lower microbial biomass compared to Standard PCR for both healthy donors. Bacterial concentration is affecting phylum and class composition for samples containing less than 10^6^ microbes (Fig. 2b), resulting in an overall decrease of Bacteroidetes and increase of Firmicutes and Proteobacteria phyla.

PCoA analysis based on Bray–Curtis distance presents two clusters characterized by sample origin (PERMANOVA, R^2^ = 0.3945, *p* < 0.001) but not by PCR protocol (PERMANOVA, R^2^ = 0.0409, *p* = 0.6174). Hierarchical cluster analysis shows that the samples containing 10^4^ and 10^5^ microbes are compositionally distant from their sample origin cluster (Fig. 2c, d). This is particularly pronounced for samples amplified with Standard PCR, for which even 10^6^ microbes (at least for donor 1) was distant from the sample origin. In fact, 10^4^ and 10^5^ biomass samples for Standard PCR and 10^4^ biomass samples for Nested PCR are distinctly clustered from their sample origin (Fig. 2c, d). This observation is further explained by a heatmap of the top 30 most represented genera, which shows that dominant species in the sample origin are largely underrepresented in low biomass biospecimens. Contrarily, minor or absent species in the sample origin appears dominant in low biomass biospecimens suggesting effects of environmental contamination (*Undibacterium*, *Blautia*, *Ruminococcaceae_UGC-013, Subdoligranulum*) (Fig. 2d). These findings are consistent with the rarefaction analysis, which demonstrate a positive association between sample biomass and both number of reads as well as ASV count (Supplementary Fig. S1a). Standard PCR resulted in lower ASV richness than Nested PCR. Especially Standard PCR of low biomass samples (< 10^6^) detected less than 30 ASVs. Moreover, comparing shared ASVs across samples with the highest microbial content sample, decreasing microbial biomass resulted in a decreased percentage of shared ASVs (range [31%;75%], Supplementary Fig. S1b). Of note, the loss of shared ASVs was explained primarily by the loss of minor taxa at all phylogenetic levels for the Standard PCR; whereas Nested PCR preserved phylogenetic resolution for almost all samples, explaining the superior performance of Nested PCR (Supplementary Fig. S2a–c).

Finally, sample similarity based on Spearman’s rank correlation of genera abundances showed that Nested PCR retained sample similarity for samples containing 10^6^, 10^7^ and 10^8^ microbes, while Standard PCR retained sample similarity for samples with 10^7^ and 10^8^ microbes (Supplementary Fig. S3).

In summary, Nested PCR analytically preserves microbiota composition better than Standard PCR when dealing with low biomass samples including 10^6^ microbes or below.

Here, we considered that large sample biomass was associated with the less biased sample composition. However, all present methodologies to determine microbiota composition suffer from inherent methodological limitations. Thus we do not know the theoretical composition of our complex gut microbiota samples. To overcome this problem, we next controlled the 16S rRNA gene sequencing quality associated with our DNA extraction and PCR protocols on a standardized microbial community.

### DNA extraction and PCR protocols impact 16S rRNA gene analysis

We assessed three DNA extraction protocols and tested different mechanical lysing programs. Each protocol was tested on microbial standards containing 8 fully characterized and equally abundant strains. The standard therefore has a maximal theoretical Shannon alpha diversity equal to log(8). Subsequently, each sample was amplified by either Standard or Nested PCR (Fig. 1b).

**Figure 3:**
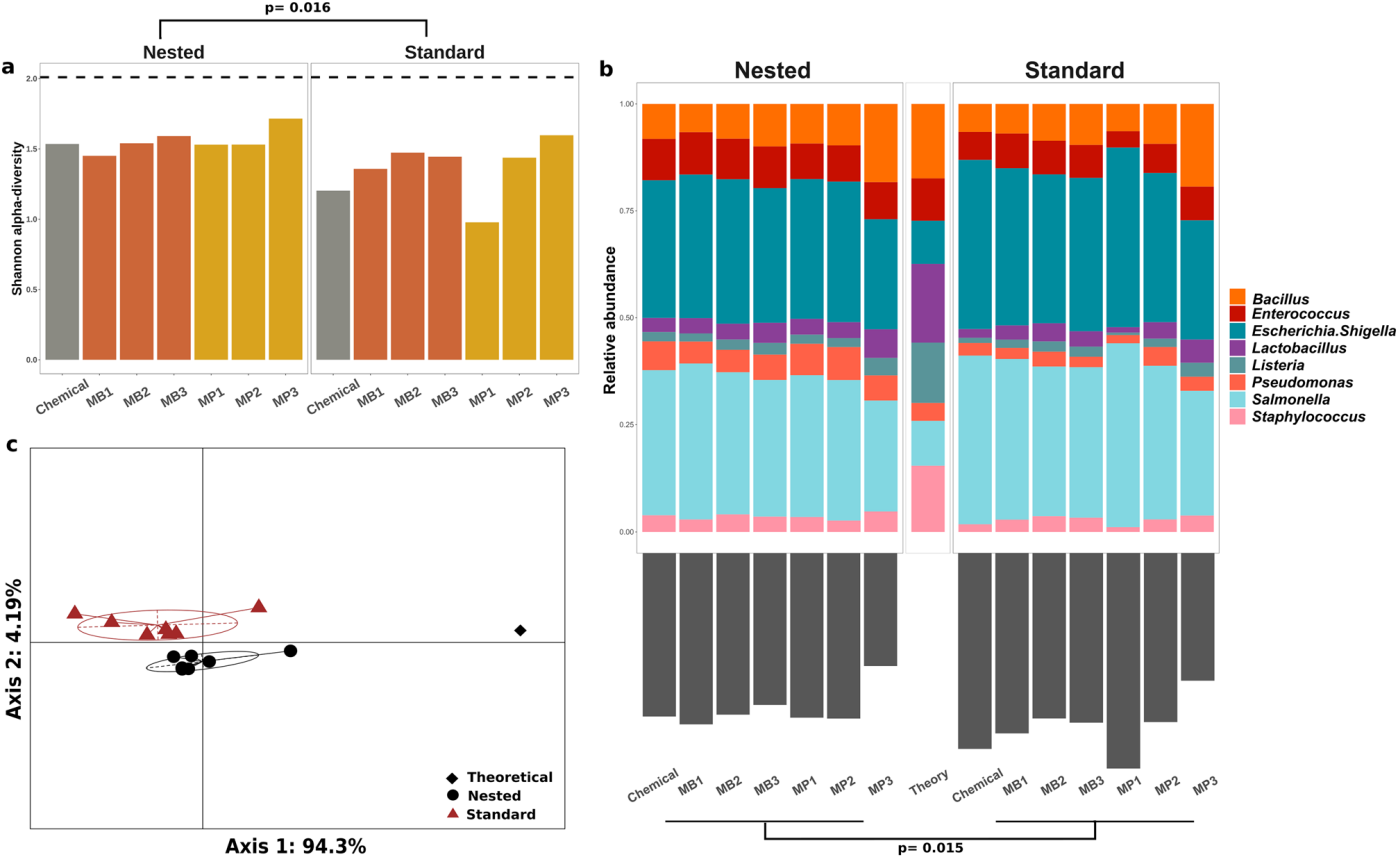
Extraction and PCR protocols effect on the representation of commercial mock microbial community standard composition. Microbial community standard was exposed to three DNA isolation methods (Chemical, Magnetic Beads (MB) and Miniprep kit (MP)), 3 mechanical lysing protocols (3 × 30 s, 2 × 5 min and 4 × 5 min, coined 1, 2 and 3, respectively) and two 16S rRNA gene PCR amplification protocols (Standard and Nested). Bacterial composition stratified according to DNA extraction and PCR was analyzed for (**a**) alpha-diversity (Dashed-line represents the theoretical Shannon index calculated with the supplier-furnished theoretical abundances), (**b**) Abundance profiles (upper panel) and Bray–Curtis distance between samples and theoretical composition (lower panel) and (**c**) Principal coordinate analysis (PCoA) based on Bray–Curtis distance discriminates between the two PCR protocols (colors). Paired statistical analysis was applied to investigate Alpha-diversity and Bray–Curtis distance differences between Standard and Nested PCR protocols (Wilcoxon test). Figures were generated using R^39^.

Alpha diversity of the mock microbial community standard shows variation across extraction and PCR protocols (Fig. 3a). Overall, alpha diversity increased with the extend of mechanical lysing. Nested PCR resulted in an increased alpha-diversity compared to standard PCR (*p* = 0.015), approaching the theoretical alpha diversity.

We measured the Bray–Curtis distance between the theoretical composition and the experimental compositions. Mechanical lysing was of great importance for the quality of extraction. Indeed, increasing mechanical lysing time and repetition improved experimental outcome demonstrated by a decreased Bray–Curtis distance to the theoretical composition (Fig. 3b). More precisely extended mechanical lysing improved the extraction (relative abundance) of bacteria such as *B. subtillis* and *L. adolescentis* (Gram + , known to be difficult to extract^29^) over *E. coli* and *S. enterica* (Gram-) (Fig. 3b). Based on the distance to the theoretical composition, we identified the optimal extraction protocol as the Miniprep kit with 4 × 5 min (MP3) of bead beating for both PCR protocols tested (Fig. 3b). PCR protocols also significantly impact the distance between experimental and theoretical composition (Fig. 3b). The Nested PCR protocol resulted in a lower Bray–Curtis distance than the Standard PCR protocol (*p* = 0.015).

Principal coordinate analysis based on β-diversity showed two distinct clusters based on PCR protocols (PERMANOVA, R^2^ = 0.261, *p* = 0.0357), but not based on extraction protocols (PERMANOVA, R^2^ = 0.098, *p* = 0.617) (Fig. 3c). Moreover, mechanical lysing time showed a statistical difference between samples (PERMANOVA, R^2^ = 0,018, *p* = 0,025). Therefore, the effect size is bigger for PCR protocols and mechanical lysing time compared to extraction protocols. Of note, the two MP3 protocols (4 × 5 min bead beating) are situated distant from their respective groups (stratified by PCR protocol), and approaching the theoretical distribution (Fig. 3c).

### PCR bias of 16S rRNA gene analysis is independent of DNA extraction bias

**Figure 4:**
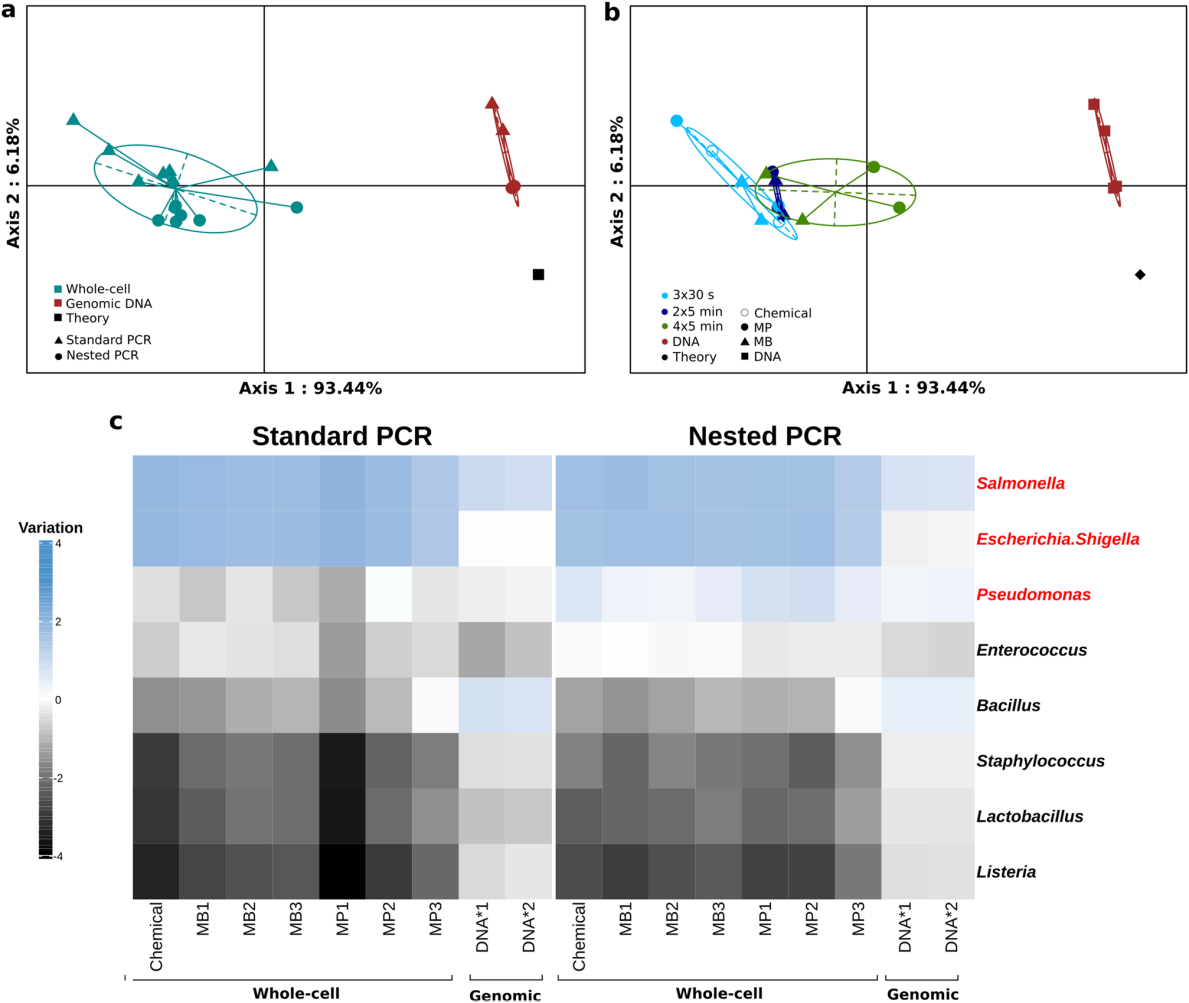
Extended mechanical lysing improves representation of gram-positive bacteria. (**a**) PCoA of whole-cell (cyan) and genomic DNA (brown) samples compared to the theoretical abundance profile (black). (**b**) The same PCoA was subjected to stratification of whole cell standards according to extraction protocol (mechanical lysing and DNA isolation). (**c**) Heatmap of variation from the theoretical abundance for all microbial community standards (columns) amplified by Standard (left panel) or Nested (right panel) PCR. The 8 bacterial taxa of the mock microbial community standard were ordered according to Gram-status; gram-negative (red) and gram-positive (black). Variation from theoretical abundance is calculated as log10[sample abundance/theoretical abundance] for each genus. Figures were generated using R^39^.

Using cellular mock microbial community standards we could identify both DNA extraction and PCR protocols as contributors of technical bias for 16S rRNA gene analysis. Whereas this approach allowed us to determine the relative bias induced by Standard and Nested PCR protocols (Nested less biased than Standard) we could not determine the absolute PCR bias because of the underlying DNA extraction bias. To eliminate DNA extraction bias we used a genomic DNA mock microbial community standard (Fig. 1c). The genomic DNA standard consisted of an equimolar mix of genomic DNA purified from the 8 individual strains also present in the cellular mock microbial community standard. Based on sample proximity to the theoretical distribution the Nested PCR protocol is superior to the Standard PCR protocol (Fig. 4a). More precisely, the Nested PCR protocol was more reproducible and resulted in better amplification for genera *Enterococcus*, *Lactobacillus*, *Pseudomonas* and *Staphylococcus* compared to Standard PCR (Supplementary Fig. S4). However, both protocols tend to over-amplify *Bacillus* and *Salmonella* genera compared to the theoretical abundance (Supplementary Fig. S4).

Multiplexed primer design (Nested PCR) and additional PCR cycles to accommodate low biomass samples are expected to produce more chimeras. We observed that Nested PCR produces more chimera sequences than Standard PCR (Wilcoxon paired test, *p* = 0.0018), but not more final sequences (*p* = 0.077) (Supplementary Fig. S5a). In the same manner, total number of observed ASVs are not different between the two protocols (*p* = 0.82, Supplementary Fig. S5b). Finally, both Nested and Standard PCR protocols resulted in very few off-target sequences (less than 0.05% relative abundance) and a maximum of two off-target sequences per sample. Adding PCR steps result in more chimera generation but these chimeras seem to be effectively handled by DADA2 chimera removal algorithm.

### Extraction bias explained by bacterial resistance to cell lysis

Hierarchical cluster analysis identifies a first principal coordinate, which segregates mock microbial community standards in two distinct clusters characterized by sample origin (cellular vs genomic community standards; PERMANOVA, R^2^ = 0.84, *p* =  < 0,001). Genomic community standard is more similar to the theoretical composition compared to the cellular community standard (Fig. 4a). Segregating by PCR protocols shows a constant pattern explained by the second principal coordinate with Nested PCR being closer to theory for both genomic and whole cell samples compared to Standard PCR (Fig. 4a). We observed that genomic DNA was much closer to the theoretical composition compared to whole cell samples irrespective of DNA extraction and PCR protocols (Fig. 4b). DNA extraction protocols with extended mechanical lysing improve microbial composition distance to theoretical abundances. This effect is accentuated using a Miniprep kit for DNA isolation. Therefore, MP3 DNA extraction protocol (Miniprep kit with 4 × 5 min mechanical lysis) combined with the Nested PCR protocol displayed close proximity between the microbial sample composition and the theoretical abundance profile (Fig. 4b). Finally, considering sample relative abundance variation from the theoretical abundance, we clearly see that gram-negative bacteria tend to be overrepresented and gram-positive bacteria underrepresented in whole-cell mock microbial community standard (Fig. 4c). Increasing mechanical lysing time is smoothing this discrepancy by improving DNA extraction of hard-to-lyse gram-positive bacteria. Removing the DNA extraction step resulted in the loss of Gram-associated discrepancies but highlighted primer performance dissimilarities as *Salmonella* and *Bacillus* tend to be over-represented, while *Enterococcus* and *Lactobacillus* tend to be under-represented (Fig. 4c). Nested PCR seems to improve this performance issue as compared with Standard PCR (*p* = 0.0004, paired T-test; Fig. 4c).

Overall, we show here that 10^6^ microbes is the lowest sample biomass which permits 16S rRNA gene analysis to respect sample composition and diversity. Indeed, DNA extraction of low biomass samples requires affinity-based DNA isolation. We show that compared to Standard PCR, our Nested PCR protocol is more sensitive, while maintaining specificity and thus robustness for samples containing 10^6^ microbes or more. Of note, 10^6^ microbes could not be accurately analyzed when amplified by Standard PCR. Extended mechanical lysing improved hard-to-lyse gram-positive bacteria representation. Finally, a synergistic effect was observed when optimizing both DNA extraction and PCR protocols.

## Discussion

Microbiome analysis despite all technological advances is affected by various methodological biases. Reducing bias from 16S rRNA gene analysis will improve data quality and our ability to compare data from different studies. Our results identify factors improving sensitivity and specificity of 16S rRNA gene sequencing. Indeed, we show that sample biomass, cell lysis, DNA isolation and PCR protocols can drastically modify 16S rRNA gene sequencing results. These confounders affect both sample diversity and composition.

Our data show that protocols developed for gut microbiota analysis cannot be transferred to low biomassmicrobiota from other body sites without prior validation. These findings are in accordance with prior studies on 16S rRNA gene analysis of serial dilutions of mock microbial community standards^11^ and genomic DNA^30^. Both studies found an important decrease in alpha-diversity and an increase in beta-diversity associated with microbial biomass. Bender et al*.* support our observation that sample biomass is the most important confounder in comparison to PCR protocols, DNA extraction or other known factors^11^. This should be considered when interpreting historic studies of low biomass biospecimens and future studies should ensure proper controls of reproducibility for such samples. Another matter regarding low biomass specimens is the contamination and its contribution to the apparent microbial community^21^, which may be accentuated in our low microbial biomass samples (10^4^ and 10^5^). Assuring repeatability and using good controls, in order to segregate contaminants from an existing microbiome within low biomass microbiota samples has previously been demonstrated^18^. Moreover, shotgun metagenomic analysis of low biomass microbiota samples is robust only for samples with more than 10^7^ microbes^23^. Here we demonstrate that 16S rRNA gene analysis by amplicon sequencing is 10 times more sensitive as it is able to generate robust results for samples with as little as 10^6^ microbes.

Efficient cell lysis is particularly important for a robust and representative microbiota analysis. Whereas Gram-negative bacteria are fairly easy to lyse, Gram-positive bacteria and fungi are increasingly difficult to lyse^29^. Therefore Gram-negative bacteria are easily overrepresented in microbiota studies compared to Gram-positive bacteria. Here we show that extended mechanical lysing improves recovery of resistant Gram-positive bacteria, such as *Staphylococcus*, *Listeria* and *Lactobacillus*. Extensive mechanical lysing is believed to drive DNA degradation^31^, which could severely bias the resulting microbiota composition. Here we do not observe such an effect even at the most extensive bead beating protocol of 4 × 5 min. Indeed, the most extensive mechanical lysing seems to better recover resistant bacteria and aligns most accurately with the theoretical microbial abundance profile.

We also observed that DNA isolation by affinity (silica columns and MagBeads, in that order) is more efficient than DNA precipitation for scarce biospecimens. DNA precipitation of low microbial biomass samples might be improved by adding a DNA/RNA carrier, but this would complexify the protocol, reduce reproducibility and was therefore not investigated further^32,33^. We demonstrated that genomic DNA isolated with silica columns could be PCR amplified for all sample biomasses tested (10^4^ to 10^8^ microbes). Genomic DNA isolated with MagBeads or chemical precipitation could not be amplified for scarce biospecimens with less than 10^6^ and 10^7^ microbes, respectively. It is not excluded that elimination of PCR inhibitors differ between protocols and play a role in the differences observed. Extraction protocols on the mock microbial community standard (10^9^ microbes) showed no major bias for the different DNA isolation methods. Here we show that a semi-nested PCR approach is superior to a standard 1-step PCR protocol. We cannot conclude here whether the apparent superiority of Nested PCR is due to differences in primer design or better sensitivity. Indeed, the absence of primer tags on the first PCR in the Nested PCR protocol (maximal sequence homology) improves primer hybridization and thus subsequent amplification compared to Standard PCR. Similar improvements were reported when adding required Illumina sequence tags in two consecutive PCR reactions for medium biomass vaginal microbiota samples^34^. Brooks et al*.* described that PCR protocols and DNA extraction affected the perceived bacterial community composition in a non-redundant manner^7^. Chimera detection and removal is a crucial step in the bioinformatic pipeline especially for complex PCR protocols. Here we show that two-step Nested PCR produce more chimera than a one-step PCR but results in no differences in terms of final reads, total observed ASVs and off-target sequences. These findings suggest that adding a PCR step is not producing additional bias and are suitable for microbiota analysis.

Our study did not address primer design. Indeed, other primer pairs targeting the same as well as other hypervariable regions of the 16S rRNA gene could improve gene amplification. Such novel primer pairs can also introduce biases and should therefore be tested on mock microbial community standards^35^. We did not assess the effect of human DNA that can be found in high quantity in some low biomass samples such as biopsies or broncho-alveolar lavages. Such contamination is known to interfere with whole genome sequencing and needs to be evaluated for 16S rRNA gene analysis of low biomass samples to validate our findings in samples contaminated with human DNA. Human DNA in high quantity can act as a DNA carrier facilitating DNA purification or impact the sensitivity of PCR amplification by competition for primer annealing. However, in silico alignment of primers against human DNA or mitochondrial 16S rRNA gene returned no hit, no mitochondrial sequences were found in our sequence data and no amplification of human/mitochondrial DNA was found with our primers using human DNA extracted from whole blood.

Finally, long-read sequencing technology will improve species coverage in comparison with short-read (< 500 bp) 16S rRNA gene sequencing^36,37^. Our findings suggest that 16S rRNA gene analysis of low biomass samples requires optimal DNA extraction procedures and a well-designed nested PCR strategy.

## Conclusion

We demonstrate that 16S rRNA amplicon sequencing as a means to describe the phylogenetic composition of a microbiota sample is affected by (1) microbial biomass, (2) DNA extraction (mechanical cell lysing and DNA isolation) and (3) PCR amplification. We show that microbial biomass is the most critical factor and only silica columns allow DNA isolation from the lowest microbial biomass samples (< 10^6^ microbes). Moreover, amplification of low microbial biomass samples required a semi-nested PCR protocol (tenfold superior to classical 1-step PCR amplification). With regards to microbial composition extended lysing protocols as well as PCR protocols affected the performance of the analysis. Of note, microbial composition analysis is also impacted by DNA isolation method. However, this effect is primarily observed for low biomass samples.

Microbiota research is rapidly moving towards more exotic anatomical body sites characterized by medium (oral, nasal, skin, vaginal, meconium) and low (lung, solid tumors (biopsies), breastmilk and placenta) microbial densities. Considering prior methodological findings^11,23,38^ as well as the observations presented here, we encourage researchers to carefully adapt their methodological approaches to low biomass microbiota samples to ensure analytical standardization and reproducibility. Our study provides an experimental protocol adapted to the analysis of low biomass biospecimens.

## Materials and methods

### Fecal sample storage

Fresh stools were collected from 2 healthy donors. Informed consent was obtained from donors in writing before study inclusion. The study design was evaluated and approved by the local ethics committee (Comité de Protection des Personnes Ile de France VI, Paris, France). Research was performed in accordance with the Declaration of Helsinki. Stools were collected immediately after emission in a container allowing anaerobic bacteria preservation (Anaerocult band; Merck, Darmstadt, Germany), placed in aliquots in a CO_2_-rich O_2_-low atmosphere and stored at − 80 °C.

### Stool purification

Fecal microbiota was extracted by means of gradient purification under anaerobic condition (Freter chamber) as previously described^39^. Briefly, 2 g of thawed feces were diluted in 1X PBS (Eurobio), 0.03% wt/vol sodium deoxycholate, and 60% wt/vol Nycodenz (Sigma-Aldrich, St Louis, Mo) and loaded on a continuous Nycodenz based density gradient obtained by a freeze–thaw cycle. Fecal bacteria were obtained after ultracentrifugation (14,567 g for 45 min at + 4 °C; Beckman Coulter ultracentrifuge, swinging rotor SW28; Beckman Coulter, Fullerton, Calif) and washed 3 times in 1X PBS (Eurobio) and 0.03% wt/vol sodium deoxycholate. The final pellet was diluted in 8 mL 1X PBS–10% glycerol, immediately frozen in liquid nitrogen, and then stored at − 80 °C^40^.

### Creation of low biomass biospecimens

Samples containing 10^8^, 10^7^, 10^6^, 10^5^ and 10^4^ microbes were prepared from serial dilutions of the two healthy donor stools. Microbial cell count was assessed using AQUIOS Flow count spheres (Beckman Coulter, B96656). Briefly, we used 1 µL of stool sample mixed with 100 µL of beads with a known concentration.

$$microbial\;concentration = \frac{{{\text{sum}}\;{\text{of}}\;{\text{cells}}\;{\text{counted}}}}{sum\;of\;beads\;counted}* beads\;concentration$$

### Microbial standards

Mock microbial community standards were purchased from Zymobiomics (Ozyme SAS, France) for whole-cell microbial community standard (D6300) and genomic DNA microbial community standard (D6305). The Zymobiomics standards contained 8 different bacterial strains at a theoretical genomic DNA concentration of 12% for each bacteria. Based on 16S rRNA gene copy numbers we obtained corrected 16S rRNA gene relative abundances of 17.4% *B. subtilis*, 9.9% *E. faecalis*, 10.1% *E. coli*, 18.4% *L. adolescentis*, 14.1% *L. monocytogenes*, 4.2% *P. aeruginosa*, 10.4% *S. enterica* and 15.5% *S. aureus*. The corrected theoretical abundance served as theoretical abundance here. Approximately 10^9^ microbes of Zymobiomics standard were used per extraction.

### DNA extraction

We employed three different DNA extraction protocols. All methods were based on chemical and mechanical cell lysis followed by DNA isolation.

Chemical extraction protocol was conducted as previously described^15^. Briefly, samples prepared as described higher were centrifugated at 12.000 rpm and (I) resuspended in guanidine thyocyanate and N-Lauroyl Sarcosine, (II) heated in a dry water bath at 70 °C followed by (III) mechanical lysis (FastPrep) using 0.1 mm and 0.5 mm glass beads. (IV) Polyvinylpyrrolidone (PVP) was used for the removal of polymerase chain reaction (PCR) inhibitors, such as polyphenols. (V) Finally, impurities are removed by precipitation in 100% isopropanol overnight. The supernatant containing the purified DNA was treated with RNAse and genomic DNA was ethanol precipitated.

For affinity-based protocols we used Zymobiomics MagBeads (Zymo Research D4306, magnetic beads) and Miniprep (Zymo Research D4300, silica column) kits. MagBeads kit was used after step IV from the chemical extraction protocol. Miniprep kit was used according to manufacturer instructions.

Mechanical cell lysis for the mock Community Standard was performed by bead beating (0.1 mm and 0.5 mm glass beads, Tissue Lyzer, QIAGEN, France) for varying amounts of time and repetitions; 3 × 30 s, 2 × 5 min and 4 × 5 min were tested. Of note, samples were left on ice for 5 min between each bead beating. Extraction of the mock microbial community standard with chemical cell lysis was conducted with 3 × 30 s mechanical lysing.

### 16S rRNA gene amplification and amplicon sequencing

The V3–V4 region of the 16S rRNA gene was amplified using either a two-step semi-nested or a one-step standard PCR protocol. These two strategies are referred to as “Nested” and “Standard” throughout the manuscript. The semi-nested PCR was performed as previously described^40^. Briefly, 16S rRNA genes were amplified using a short pre-amplification of 10 cycles with primer couple S-D-Bact-0343-a-S-15 (+ 343; 5′ TACGGRAGGCAGCAG 3′) and S-D-Bact-0907-a-A-20 (+ 907; 5′ CCGTCAATTCMTTTRAGT 3′) (annealing temperature: 54 °C) followed by a second amplification with 40 cycles of standard PCR. The standard PCR was conducted with the primer couple S-D-Bact-0343-a-S-15_GenoToul(+ 343, 5′CTTTCCCTACACGACGCTCTTCCGATCTACGGRAGGCAGCAG 3′) and S-D-Bact-0784-a-A-15 _GenoToul(+ 784; 5′GGAGTTCAGACGTGTGCTCTTCCGATCTTACCAGGGTATCTAATCCT 3′) for the GenoToul platform (see Library preparation) and S-D-Bact-0343-a-S-15_biofidal (+ 343, 5′ TCGTCGGCAGCGTCAGATGTGTATAAGAGACAGACGGRAGGCAGCAG 3′) and S-D-Bact-0784-a-A-15_biofidal (+ 784, 5′ GTCTCGTGGGCTCGGAGATGTGTATAAGAGACAGTACGAGGGTATCTAATCCT 3′) for the BioFidal platform (annealing temperature of 62 °C). The Standard PCR primers combine 16S rRNA gene-specific primers with MiSeq adapter sequences (underlined). Amplification reactions were performed with DNA MolTaq (Molzym, Bremen, Germany). Multiplexing was accomplished by adding index k-mers to the amplicons. The genotoul platform used a 6-mer index, which was added to the 3′ end of the amplicon through a 12 cycle PCR reaction using forward primer (AATGATACGGCGACCACCGAGATCTACACTCTTTCCCTACACGAC) and reverse primer (CAAGCAGAAGACGGCATACGAGAT-6 bp index-GTGACTGGAGTTCAGACGTGT). The Biofidal platform added 15-mer indexes to both 5′ (i5) and 3′ (i7′) ends of the amplicon through a 10 cycle PCR reaction using forward primer (AATGATACGGCGACCACCGAGATCTACAC[i5]TCGTCGGCAGCGTC) and reverse primer (CAAGCAGAAGACGGCATACGAGAT[i7′]GTCTCGTGGGCTCGG). PCR amplicon libraries were sequenced on a MiSeq Illumina platform (Genotoul, Toulouse, France and Biofidal, Vaulx-en-Velin, France) producing 2 × 300 bp paired-end reads. The quality of the sequence run was checked internally using PhiX, and then each paired-end sequence was assigned to its sample with the help of the previously integrated index.

### Bioinformatics for 16S rRNA gene analysis

Further processing of demultiplexed sequence reads followed the DADA2 workflow for Big Data (https://benjjneb.github.io/dada2/bigdata.html26) and employed R software (version 4.0.4^39^) and the DADA2 package (v. 1.5.2). Briefly, sequences were quality filtered, trimmed and assembled. Lengths of 240 bp and 240 bp were chosen for hard trimming of forward and reverse reads, respectively, because these were the lengths beyond which median quality scores decreased below 20 for the lowest-quality library and to ensure that paired sequences contained sufficient information for merging (overlapping 3′ ends). Individual reads were further truncated at the base, where a quality score of 2 was observed, and filtered to contain no ambiguous bases. Additionally, the maximum number of expected errors in a read was set to 2. Reads were assembled only if the overlap between forward and reverse reads, which occurs in the conserved region between V3 and V4, was 100% identical. Chimeras from combined runs were removed by the DADA2 protocol. Amplicon sequence variants (ASVs) generated by DADA2 analysis of the quality-filtered sequence data were taxonomically classified using the RDP naïve Bayesian classifier^41^, trained with the Silva 16S rRNA gene taxonomic training data formatted for DADA2 (Silva version 132; https://zenodo.org/record/1172783/files/silva_nr_v132_train_set.fa.gz). Read counts for ASVs assigned to the same taxonomy were summed for each sample. For the 18 serial dilutions samples of the 2 healthy donor stools included in our study we obtained 881,881 paired-end reads (average: 48,993 reads/sample), which were reduced to 287,332 reads with appropriate phylogenetic assignment (average: 15,963 paired-end reads/sample). We equally included 18 mock microbial community standard samples, for which we obtained 782,703 paired-end reads (average: 43,484 paired end reads/sample), which were reduced to 254,409 phylogenetically assigned paired-end reads (average: 14,134 paired-end reads/sample). Our negative control resulted in 8274 paired-end reads, which were reduced to 53 phylogenetically assigned paired-end reads. Three normalization protocols were applied to the sequences as described in the result section. Figures represent analysis with rarefaction at a maximum of 10,000 reads. Samples with less than 10,000 reads were conserved in full.

### Data analysis

Microbiome composition was studied with the Phyloseq R package (version 1.28). Alpha diversity was assessed as the Shannon diversity index and beta-diversity as the Bray–Curtis distance. The distance matrix was subjected to a Principal Coordinate Analysis (PCoA). PERMANOVA test was conducted in the vegan R package (2.5.6) with the *adonis* function. Statistical analysis was performed in R (version 3.6.1) using non-parametric Mann–Whitney Wilcoxon test (paired and unpaired), parametric Student paired test and Spearman correlations. Heatmaps were generated with ComplexHeatmap R package (2.0.0). Plots were generated using ggplot2 (3.2.1), corrplot (0.84) and ade4 (1.7.13). Primers pairwise alignment against mitochondrial 16S rRNA gene and other single alignments were performed using r package Biostrings (2.58.0). Figures were assembled using Inkscape software.

### Ethics approval and consent to participate

Informed consent was obtained from all study individuals and the protocol was approved by the local ethical committee of the Pitié-Salpêtrière hospital.

## Supplementary Information

Supplementary Information 1.

## Data Availability

R script is available on www.immulab.fr/cms/index.php/team/publications-suppl. Data are available under accession number PRJEB44893 in the European Nucleotide Archive (ENA).
