# Supplementary material for: Refinement of 16S rRNA gene analysis for low biomass biospecimens
Source: Sci Rep. 2021 May 24;11:10741. doi: 10.1038/s41598-021-90226-2 (PMC8144411; doi:10.1038/s41598-021-90226-2)
Supplement: Supplementary file 1 — Supplementary Information 1. [file 41598_2021_90226_MOESM1_ESM.pdf]

Refinement of 16S rRNA gene analysis for low biomass biospecimens.

Remy VILLETTE[1*], Gaelle AUTAA[1*], Sophie HIND[2], Johanna B HOLM[3], Alicia MORENO-SABATER[1,2,4] and Martin LARSEN[1,2,‡]

[1]Inserm UMR-S1135, Centre d'Immunologie et des Maladies Infectieuses (CIMI-Paris), F-75013 Paris, France.

[2]Sorbonne Universités, UPMC Univ Paris 06, CR7, Centre d'Immunologie et des Maladies Infectieuses (CIMI-Paris), Hôpital Pitié-Salpêtrière, 83 bd. de l'Hôpital, 75013 Paris, France.

[3]Institute for Genome Sciences and Department of Microbiology and Immunology, University of Maryland School of Medicine, Baltimore, Maryland, USA.

[4]Département d'Immunologie, AP-HP, Groupement Hospitalier Saint Louis, F-75010 Paris, France.

[*]Contributed equally

Supplementary figures

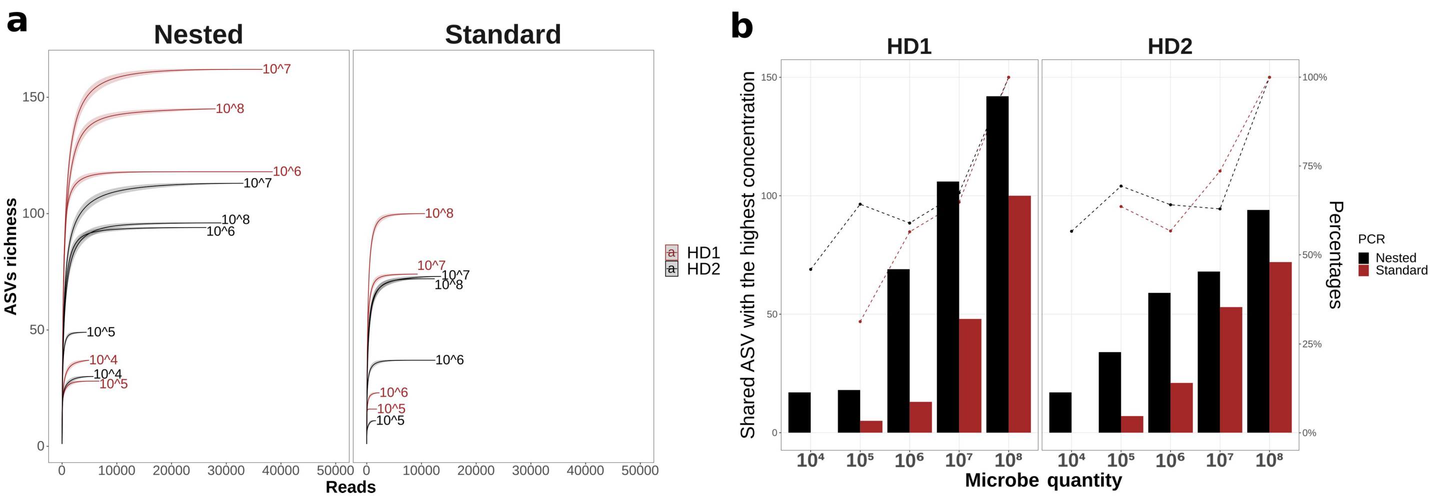

Supplementary figure 1: Nested PCR retains more ASVs than Standard PCR. a Rarefaction curve of healthy donor samples containing 10^8, 10^7, 10^6, 10^5 and 10^4 microbes, respectively. **b** Shared ASVs between the higher microbial biomass (10^8 microbes) and the others (10^4-10^7 microbes) in absolute numbers (first axis) and in percentage (second axis).

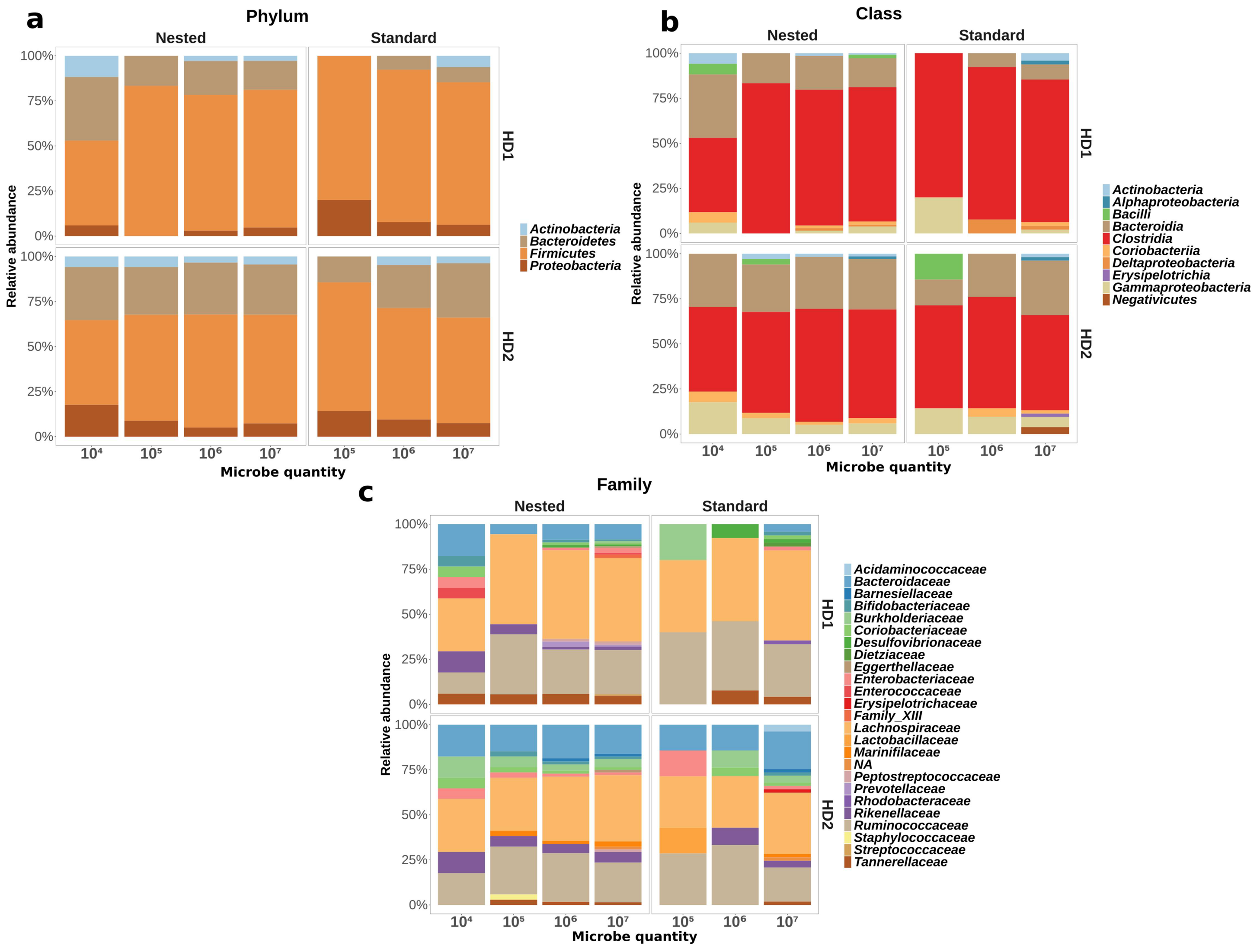

Supplementary figure 2: Nested PCR retains more phylogenic information than Standard PCR. Relative abundance of shared ASVs between 10^8 microbes and lower biomass biospecimens (10^4-10^7 microbes) at **a** phylum level, **b** class level and **c** family level.

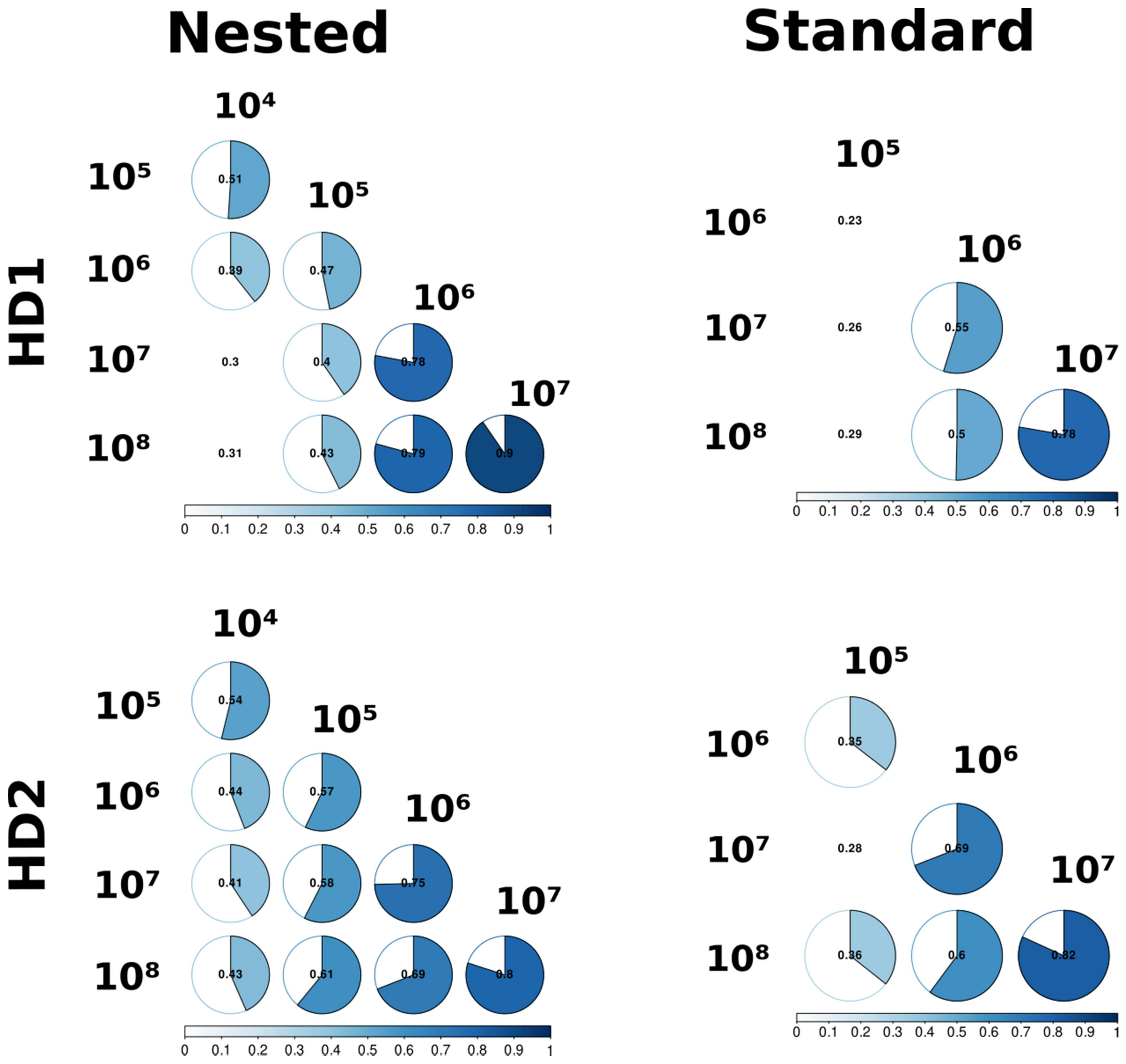

Supplementary figure 3: Loss of strong correlation below 10^6 microbes. Correlogram between healthy donor samples containing 10^8, 10^7, 10^6, 10^5 and 10^4 microbes. Pie chart are displayed only for significant correlations.

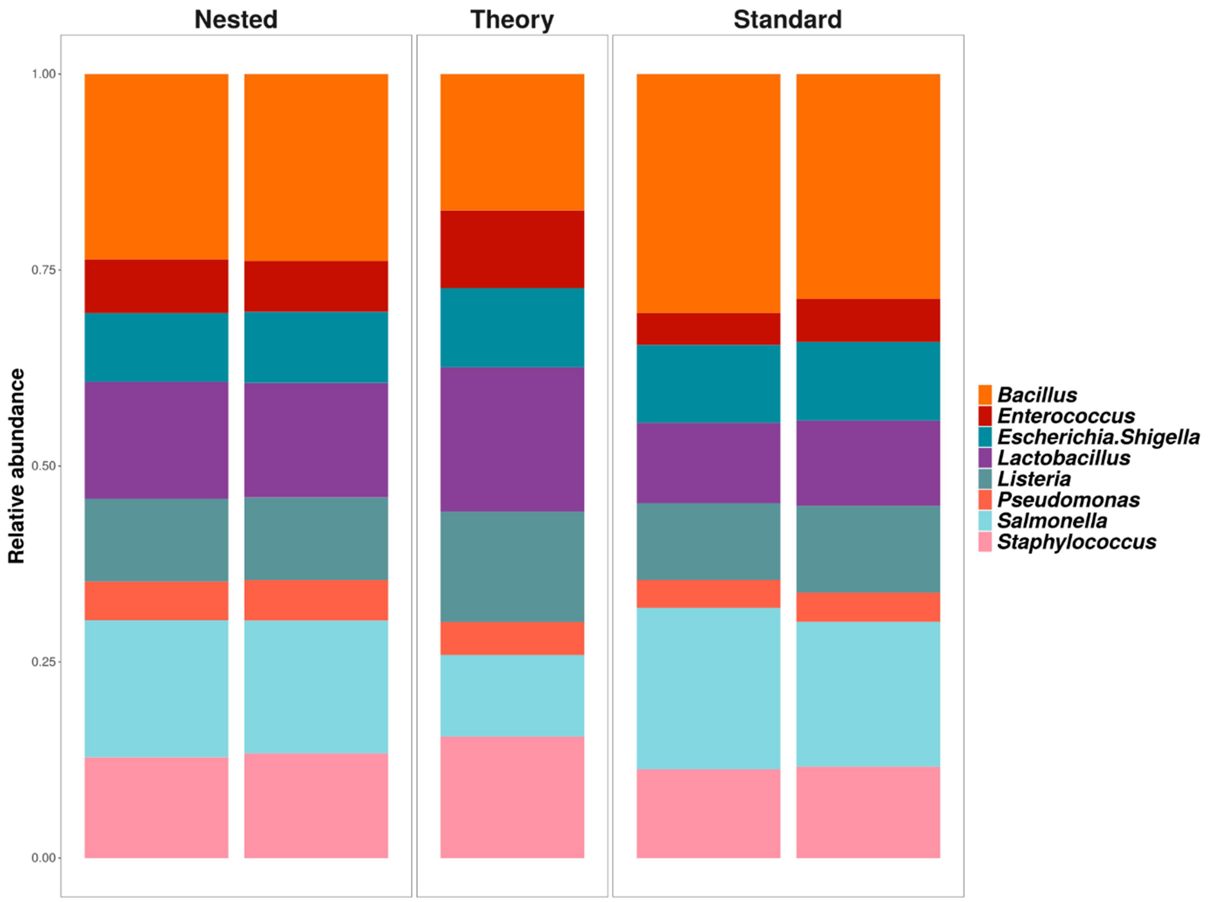

Supplementary figure 4: Nested PCR recovers slightly better the relative abundance and microbial profile of DNA mock community standard than Standard PCR. Relative abundance of DNA mock community standard amplified by Nested PCR protocol (duplicate) and Standard PCR protocol (duplicate). Middle panel represent the theoretical abundance normalized according to 16S copy number.

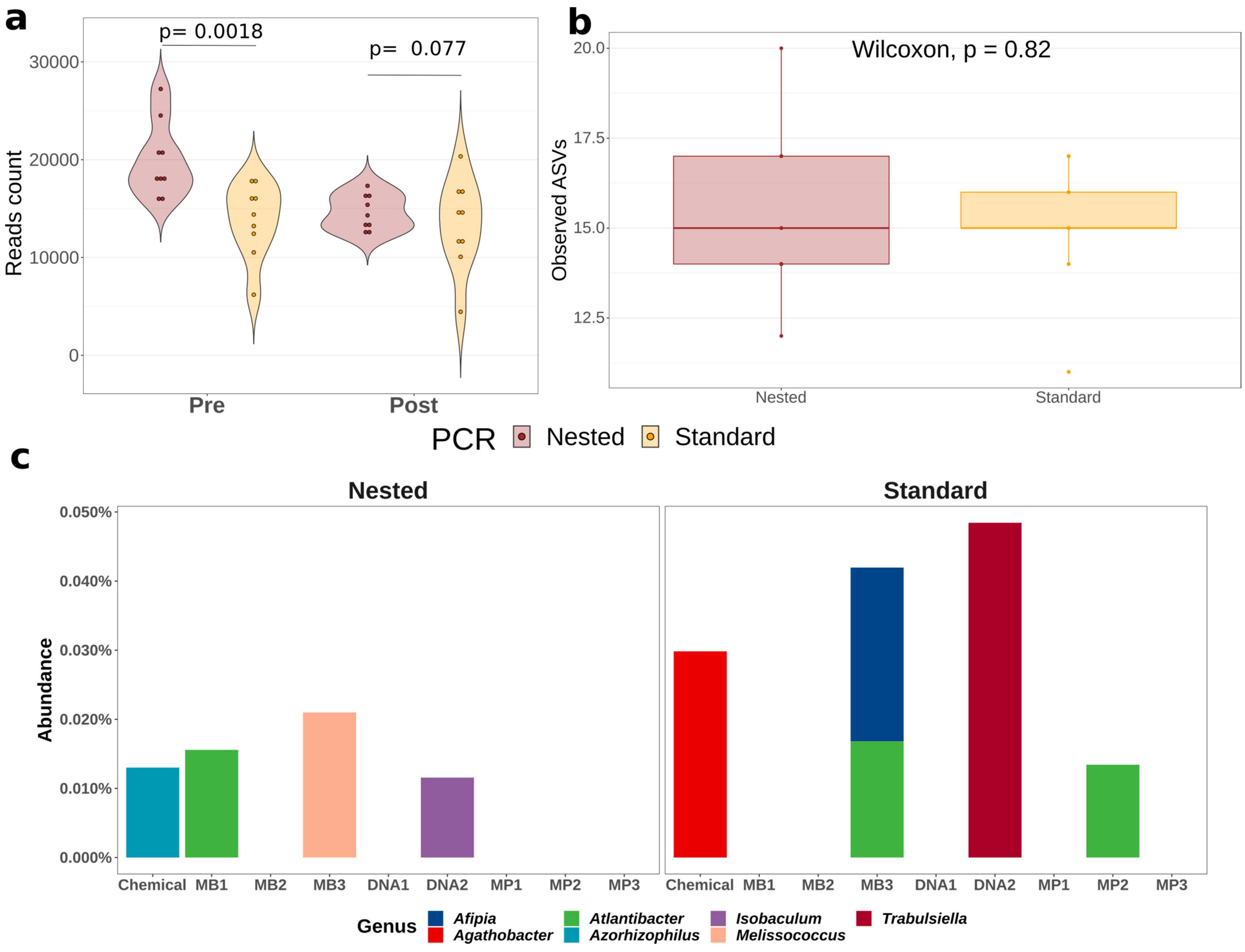

Supplementary figure 5: Two-step PCR does not produce more fortuitous sequences than a classical PCR. a Read counts pre and post chimeras removal and **b** total observed ASVs for mock community standards analyzed with Nested and Standard PCR protocols. **c** Detection of fortuitous sequences in mock community standards. Fortuitous sequences were defined as sequences not belonging to genera present in the MCS composition. MB refers to MagBeads protocol, MP refers to Miniprep protocol and numbers refer to lysing time of 3x30s, 2x5m and 4x5min. DNA refers to genomic mock community standard replicate one and two. Statistical comparison was conducted with a Wilcoxon paired test.
